# *Limosilactobacillus reuteri* promotes melatonin release from human intestinal organoids via 5′ectonucleotidase activity

**DOI:** 10.1080/19490976.2026.2670854

**Published:** 2026-05-18

**Authors:** Micah D. Forshee, Erika J. Nachman, Esha R. Shenoy, Heather A. Danhof, Ludwig Ermann Lundberg, Stefan Roos, Robert A. Britton

**Affiliations:** a Department of Molecular Virology & Microbiology, Baylor College of Medicine, Houston, TX, USA; b BioGaia AB, Stockholm, Sweden; c Department of Molecular Sciences, Uppsala BioCenter, Swedish University of Agricultural Sciences, Uppsala, Sweden; d Alkek Center for Metagenomics and Microbiome Research, Baylor College of Medicine, Houston, TX, USA; e Dan L. Duncan Comprehensive Cancer Center, Baylor College of Medicine, Houston, TX, USA

**Keywords:** *Limosilactobacillus reuteri*, DSM 17938, DSM 32846, BG-R46®, probiotic, melatonin, human intestinal organoid, pediatric organoid, ectonucleotidase, adenosine, zinc, carbon utilization, sucrose, raffinose, stachyose, maltose, infant colic

## Abstract

Strains of *Limosilactobacillus reuteri* have been used to prevent or treat various conditions; however, the mechanisms by which they exert beneficial effects are not completely understood. Infant colic is one example in which *L. reuteri* DSM 17938 reduces clinical symptoms. While the etiology of colic is unknown, abnormal melatonin levels in infants have been suggested as a possible contributor. *L. reuteri* DSM 17938 has been shown to produce adenosine from AMP via production of the extracellular enzyme 5′ectonucleotidase (5′NT). Adenosine is a potent signaling molecule that impacts several important aspects of host physiology, including the release of melatonin from the pineal gland in the brain. A second major source of melatonin production is enteroendocrine cells in the intestine. We hypothesized that the adenosine generated via the 5′NT activity of *L. reuteri* DSM 17938, would stimulate melatonin release from human intestinal organoids. Here, we characterized the growth conditions that impact *L. reuteri* DSM 17938 5′NT activity, including carbon source utilization and required metal cofactors. We found zinc to be an essential cofactor for 5′NT activity by *L. reuteri* and observed carbon utilization altered 5′NT activity levels. Stachyose and raffinose increased levels of 5′NT activity while sucrose decreased 5′NT activity. We demonstrated that *L. reuteri* DSM 17938 stimulates melatonin release from pediatric human intestinal organoids in a 5′NT-dependent manner. Surprisingly, adenosine was necessary, but not sufficient, for the induction of epithelial melatonin release, thereby suggesting that an additional secreted factor was also required. Furthermore, *L. reuteri* BG-R46^®^, an evolved strain of DSM 17938 that is known to express higher 5′NT activity, was shown to induce higher levels of melatonin secretion. Taken together, this work identifies zinc and carbon sources as key factors altering *L. reuteri* 5′NT activity levels and demonstrates that the *L. reuteri* strains stimulate intestinal melatonin release via 5′NT.

## Introduction

Over the past few decades, leveraging beneficial intestinal microbes to improve host outcomes has received increased attention. The human probiotic strain *Limosilactobacillus reuteri* DSM 17938, formerly *Lactobacillus reuteri,*^1^ has been shown to exert positive effects on host health,^2-4^ though the mechanisms are often unknown. One specific example of this is *L. reuteri* DSM 17938 reduction of infant colic symptoms. Meta-analyses of clinical trials have shown that *L. reuteri* DSM 17938 is effective in accelerating the resolution of infant colic,^5^^,^^6^ which is defined as crying three or more hours a day at least three days a week in an otherwise healthy infant.^7^ These symptoms can lead to distress in the parent/child bond^8^ and may increase the risk of irritable bowel syndrome later in life.^9^ Studies have also linked infant colic symptoms with factors such as intestinal inflammation^10^ and melatonin levels.^11^^,^^12^

Melatonin is widely known for its activity in the brain, yet other physiological sites harbor melatonin, such as breast milk^13^ and the gastrointestinal tract.^14^ Beyond sleep and circadian rhythms, melatonin plays an important physiological role as an anti-inflammatory molecule,^15^ an antioxidant,^16^ a visceral pain reducer,^17^ and as a modulator of intestinal motility.^18^ Within the intestinal epithelium, there exist hormone-producing cells called enteroendocrine cells. Enterochromaffin cells are a subtype of enteroendocrine cells that produce and secrete melatonin^14^^,^^19^ and its precursor serotonin.^20^ However, very little is known about the molecular regulation of melatonin synthesis and release in the intestinal epithelium.

The gastrointestinal microbiome modulates the secretion of many enteric hormones, yet much remains to be learned about how the microbiome impacts gut melatonin secretion. One clinical trial investigated the effects of probiotics and melatonin on irritable bowel syndrome outcomes. In this work, supplementation with the probiotic cocktail VSL#3 improved the pain score in patients and significantly increased the amount of detectable melatonin in the saliva of males.^21^ Song and colleagues demonstrated that *Roseburia hominis* promoted melatonin release from a neuroendocrine tumor cell line.^22^ Additionally, a recent study using mouse models demonstrated that bacterial supplementation increased the abundance of melatonin's rate-limiting enzyme in the colonic epithelium. After a week of bacterial supplementation, melatonin levels were elevated in the colon but not in the serum.^23^ However, it is unknown whether bacteria can directly stimulate melatonin secretion from the human intestinal epithelium.

Like other intestinal hormones, much more work has been done characterizing the molecular regulation of melatonin production and secretion in the brain than in the gut. In the pineal gland, an important regulator of melatonin secretion is adenosine,^24^^,^^25^ which is also a potent anti-inflammatory immune signal.^26^ Interestingly, *L. reuteri* DSM 17938 has been observed to increase the levels of adenosine^27^ and the downstream adenosine metabolite inosine^27^^,^^28^ in the plasma of mice. Furthermore, in a mouse model of autoimmunity, oral gavage of *L. reuteri* improved mouse survival,^28^ but the beneficial effect was lost when the host adenosine receptor A2A was knocked out.^29^

One potential source of adenosine in the gut is bacterial 5′ectonucleotidases (5′NTs). 5′NTs are enzymes capable of cleaving adenosine monophosphate (AMP) into adenosine and inorganic phosphate. *Streptococcus* and *Staphylococcus* are common gut bacteria that generate adenosine via this mechanism to dampen the host immune response and promote bacterial survival.^30^^,^^31^ While *L. reuteri* DSM 17938 and BG-R46^®^, an evolved strain derived from DSM 17938, possess a 5′NT enzyme on extracellular membrane vesicles;^32^ its role in bacterial physiology and intestinal melatonin stimulation is unknown. Based on these results, we hypothesized that *L. reuteri* DSM 17938 can stimulate melatonin release from the intestinal epithelium via adenosine signaling, which is dependent on 5′NT activity. This study aimed to provide evidence of direct bacterial stimulation of melatonin release from the human intestinal epithelium and to investigate the mechanism by which *L. reuteri* strains can stimulate intestinal melatonin release. We also sought to determine nutrients that can impact 5′NT activity levels to obtain a better understanding of additional factors that can inform future studies to maximize the impact of *L. reuteri* DSM 17938 on colic.

## Methods

### *L. reuteri* strains

Three strains of *Limosilactobacillus reuteri* were used in this study (Supp. Table S1). We obtained *L. reuteri* DSM 17938^33^ and DSM 32846^34^ (commercial name BG-R46^®^, trademark of BioGaia AB), from BioGaia. An *L. reuteri* DSM 17938 mutant derivative lacking the 5′NT gene,^35^ Δ*sdpA,* (∆5′NT) was a generous gift from the van Pijkeren Lab at the University of Wisconsin-Madison.

### Bacterial culture conditions

About 10 mL of De Man-Rogosa-Sharpe broth (MRS) (BD Difco) was inoculated from *L. reuteri* glycerol stocks and incubated at 37 °C overnight (~16 h). Experimental supernatants were generated by subcultures with a starting optical density at 600 nm (OD_600_) of 0.1 for time intervals as indicated in the text at 37 °C overnight in a water bath. Media preparations include MRS, chemically defined LDM4,^36^ ZMB1^37^ media, or LDM4 media lacking glucose and supplemented with 20mg/mL D-(+)-glucose “glucose” (Sigma-Aldrich SLCQ7550), sucrose “sucrose” (Fisher BP220-1), stachyose hydrate “stachyose” (Chem Impex 32400), D-(+)-raffinose pentahydrate “raffinose” (Thermo Scientific A18313.09), or D-(+)-maltose monohydrate “maltose” (Alfa Aesar A16266.36). Some LDM4 preparations also received zinc supplementation at the concentrations indicated in the text. The recipes for the individual components of LDM4 and final composition of LDM4 can be found in Supplemental Tables S2 and S3, respectively. Bacterial cells were collected by centrifugation at 4000 × *g* for 5 min, and the cell-free supernatant was stored at −20 °C for downstream assays.

### Preparation of industrially produced freeze-dried bacteria

Samples with industrially produced powder with freeze-dried DSM 17938 (three batches) and BG-R46^®^ (two batches) intended for commercial products and clinical studies were prepared for organoid experiments. 0.2 g of powder (containing approximately 10^11^ cfu/g) was suspended and rehydrated in 10 mL of PBS, followed by centrifugation at 4000 × *g*, pH neutralization using 0.1 M NaOH, and sterile filtration through a 0.2 μm filter. A control solution resembling the lyoprotectant used for the industrial batches was prepared and sterile filtered (0.2 μm filter).

### *L. reuteri* DSM 17938 and Δ5′NT 24-h growth curves

To assess growth, MRS or LDM4 subcultures were prepared in Hungate tubes, and the cell density was monitored by OD_600_ at the indicated intervals (Thermo Scientific Genesys 20). ODs were directly measured in Hungate tubes or in a disposable cuvette after dilution was necessary. Doubling times were calculated via an interpolated quadratic equation (GraphPad Prism version 10) using points during exponential growth. Points between 3 and 6 h were used for MRS cultures, while points between 3 and 8 h were used for LDM4 cultures.

### Quantification of 5′NT activity

In parallel with the growth curves in MRS and LDM4, 125 µL of culture was sampled at 6, 12, and 24 h to assess 5′NT activity. 5′NT activity was quantified per the manufacturer's instructions using a colorimetric 5′-Nucleotidase Assay Kit (Crystal Chem Cat #80229) as previously reported.^32^^,^^38^ The kinetic assay was read at 3 min and 60 min (Tecan Infinite F200 Pro). Two technical replicates were averaged for each biological replicate. Samples that had values below the assay's limit of detection of 0 were given values of 0.

### Growth of *L. reuteri* DSM 17938 with various metals

To determine the nutrient requirements for 5′NT activity, LDM4 subcultures of *L. reuteri* DSM 17938 were supplemented with 17.4 μM of either zinc sulfate heptahydrate “zinc” (Sigma-Aldrich Z4750), copper(II) chloride “copper” (Sigma-Aldrich 203149), or cobalt(II) chloride hexahydrate “cobalt” (Sigma-Aldrich C8661). Dose dependency of zinc for 5′NT activity was assessed between 0 μM and 1,740  μM, as indicated. Removal of zinc from the medium was achieved by the addition of 30 μM *N*, *N*, N′, N′-Tetrakis (2-pyridymethyl) ethylenediamine “TPEN” (Sigma-Aldrich P4413) to the medium with 20 μM zinc. About 220 μM of zinc was used to quench TPEN activity. At 6 h of growth, the bacterial density was assessed by OD_600_, and the supernatant was collected for 5′NT quantification as described above.

### Assessment of carbon source utilization by *L. reuteri* DSM 17938

To determine the carbon sources that support *L. reuteri* DSM 17938 growth and/or 5′NT activity, we utilized Biolog Phenotype MicroArray 96-well plates for PM1 and PM2A. Overnight cultures of *L. reuteri* DSM 17938 were diluted to an OD_600_ of 0.1 in LDM4 media with 200 μM zinc (LDM4z200) without glucose, and 100 μL of cell suspension was added to each well. Plates were sealed with an optically clear film (VWR Cat#60941-070) and incubated aerobically for 10 h with an OD_600_ reading taken every 15 min after 5 s of shaking (Biotek Cytation 5 or Synergy H1). Total growth of each well was determined by max OD_600_—max OD_600_ (no carbon). Each biological replicate is an independent assay. Following growth measurements, the cells were collected via centrifugation, and the supernatants were assayed for 5′NT activity as described above.

### Human intestinal organoid cultivation and stimulation of melatonin secretion

Human intestinal organoid (HIO) lines from the jejunum were obtained from the Gastrointestinal Model Systems Core at the Baylor College of Medicine. Organoid cultures were maintained in 3D culture as previously described.^39^^,^^40^ Assays were performed in three genetic backgrounds. Two pediatric jejunal organoid lines from different donors (cell lines J1005 and J1006)^39^ and a transduced adult organoid line, with doxycycline-inducible overexpression of *NGN3*, were enriched for hormone-secreting enteroendocrine cells.^40^ HIO monolayers were generated following a previously published protocol.^40^ In brief, 3D organoids were disbursed into single-cell suspensions and transferred to a Matrigel (Corning, Product #354248) coated flat-bottom 96-well plate (Costar Ref. 3595, Corning) and incubated in proliferation medium at 37 °C with 5% CO_2_ to generate 2D cell monolayers for melatonin secretion assays. About 24–48 h later, the proliferation medium was replaced with differentiation medium (Supplemental Table 4), resulting in 95%–100% confluent 2D monolayers. Monolayers from the *NGN3* line received 1 µg/mL doxycycline to stimulate the increase of enteroendocrine cells during the 4 d of differentiation. Pediatric organoids were also differentiated for four days prior to experimental assays. The composition of the differentiation medium can be found in Supplemental Table 4.

Supernatants from *L. reuteri* DSM 17938 and *L. reuteri* Δ5’NT strains were made in LDM4 plus 20 μM zinc (LDM4z20) and cultured for 5‒7 h at 37 °C. Cells were collected via centrifugation at 4000 × *g* for 5 min. Supernatant was removed, the pH was neutralized (~7.0), and sterilized through a 0.22 µm PVDF syringe filter (Millex-GV) and stored at −20 °C until the assay was performed. A 1/10 by volume of 20 mM adenosine monophosphate solution “AMP” (Fisher Scientific Cas#61-19-8) was added to the thawed supernatant (final concentration of 2 mM AMP), or a 1/10 by volume of sterile distilled H_2_O was used as a control, and incubated for 30 min at 37 °C to equilibrate the temperature. Differentiation medium was removed from the monolayers prior to the addition of the prewarmed supernatants. Prewarmed supernatants were added to two monolayer wells per condition. The organoid monolayers were then incubated for 3 h at 37 °C in 5% CO_2_. After incubation, the organoid-conditioned media were removed and stored at −20 °C until later analysis. Organoid-conditioned media were quantified for melatonin with the Melatonin Serum ELISA (Eagle Bioscience MEL31-K01) per the manufacturer's instructions. Values below the limit of detection were given values equal to half of the lowest standard, which equals 1.5 pg/mL. A biological replicate is composed of the average of two wells from a single organoid plate. All melatonin secretion experiments followed the general setup as described above, except where otherwise noted.

Experiments with different carbon sources were cultured as previously described. Supernatants were collected from cultures grown for 24 h with 200 μM zinc. Prewarmed supernatants were combined with a 1/10 volume of 20 mM AMP, resulting in a final concentration of 2 mM AMP or with a 1/10 volume of water, and immediately added to the organoid monolayers, followed by 15 min of incubation at 37 °C and 5% CO_2_ rather than 3 h. Experiments with supernatants that were size fractionated underwent the following additional steps: *L. reuteri* DSM 17938 was cultured in LDM4z200 for 24 h at 37 °C, neutralized with pH, and fractionated through a 3 kilodalton (kD) filter (Amicon Ultra—4 Centrifugal filters REF# UFC800324 or UFC800396) by centrifugation for 30 min at 4000 × *g*. The flow through (size < 3 kD) and the retentate (size > 3 kD) were diluted back to their starting volumes with phosphate-buffered saline (PBS). Fractions were filter sterilized and stored as above.

Additional steps, when indicated, were performed immediately prior to the assay. After thawing, the fractionated supernatants were incubated with a final concentration of 2 mM adenosine (Sigma-Aldrich A9251) or water for 30 min at 37 °C. For some experiments, fractionation was repeated for the >3 kD fraction with adenosine. The <3 kD flow through and the >3 kD retentate were diluted back to half the original volume with PBS, 500 µL of each was combined, and then the rest of the flow through and retentate were diluted back to the original volume with PBS. Treated pediatric organoids were incubated for 1 h at 37 °C with 5% CO_2_ with the fractioned supernatants, and melatonin was quantified from the conditioned media as above.

To test whether adenosine or inosine alone was able to stimulate melatonin, LDM4z200 medium was combined with 1/10 by volume of water, 20 mM adenosine, or 20 mM inosine (Sigma-Aldrich I4125). Final concentrations of adenosine and inosine were both 2 mM. Pediatric organoids were treated and incubated for 1 h at 37 °C with 5% CO_2_ before the conditioned media were removed for melatonin quantification as described above.

To test whether commercial preparations of *L. reuteri* DSM 17938 and BG-R46^®^ retained the ability to stimulate intestinal melatonin secretion, the supernatants received from BioGaia were diluted 1/10 by volume with 20 mM AMP (final concentration of 2 mM AMP) or with water. A lyoprotectant control was also received that was diluted with 1/10 by volume with 20 mM AMP or water. Pediatric HIOs were treated with these prepared supernatants and lyoprotectant control and then incubated for 15 min at 37 °C, 5% CO_2_ before the conditioned media were removed for melatonin quantification as described above. To control for the sugar used as a lyoprotectant, values from a lyoprotectant control were subtracted from the values obtained from the batches of each strain. Supernatants were received from BioGaia in a blinded fashion, and the results were unblinded by BioGaia after the experiments were completed.

### Statistical analysis

Statistical analyses were conducted with GraphPad Prism (version 10) software, as indicated in the figure legends. When only two samples were compared, Student's *t* test was used. One-way ANOVAs were followed by multiple comparisons with Dunnett's correction when appropriate. Two-way ANOVAs were followed by multiple comparisons with either Tukey's correction or uncorrected Fisher's LSD when appropriate. Multiple unpaired *t* tests were followed by two-stage step-up (Benjamini, Krieger, and Yekutieli) corrections. *p* values < 0.05 were considered significant. Data are presented as the means ± standard deviations.

## Results

### *L. reuteri* 5′NT activity is dependent on growth conditions

**Figure 1.**
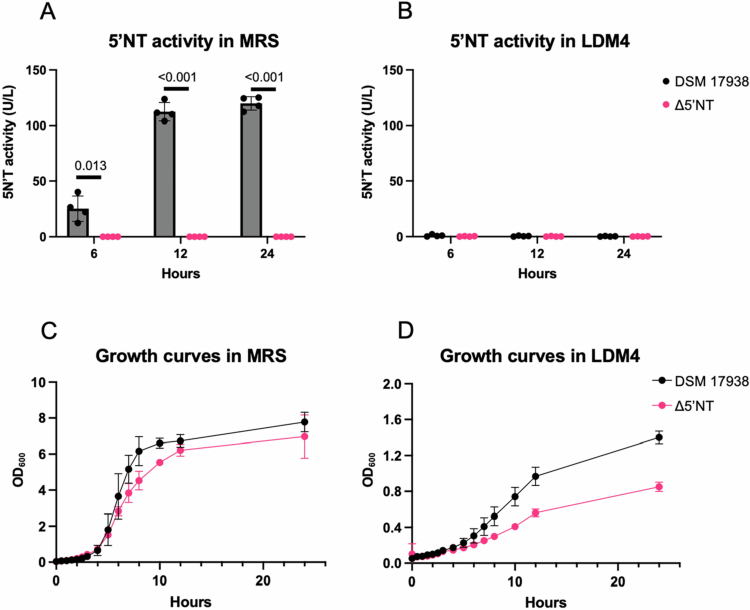
5′ectonucleotidase activity and growth curves of *L. reuteri DSM* 17938 and Δ5′NT strains grown in MRS and LDM4. *L. reuteri* DSM 17938 and Δ5′NT were grown for 24 h in MRS and LDM4. The 5′-ectonucleotidase activity was quantified in the bacterial supernatants in both MRS (A) and in LDM4 (B). Growth was measured over the 24-h growth period by measuring the optical density (C, D). Doubling times of exponential growth for both strains were calculated for growth in MRS (C) and in LDM4 (D) with *L. reuteri* Δ5′NT having reduced doubling time in both media. Student's *t* test was used to determine the statistical significance of differences between the 5′NT activity of *L. reuteri* DSM 17938 and Δ5′NT strains at different timepoints in A and B. *N* = 4 biological replicates.

Previously, 5′NT activity of *L. reuteri* DSM 17938 was assessed in rich, complex MRS media.^32^^,^^41^ Because MRS is toxic to human intestinal organoid (HIO) cultures, we used the chemically defined medium LDM4, which has been used previously for testing *L. reuteri* supernatants in HIO experiments.^42^ *L. reuteri* strains (Supp. Table S1) DSM 17938 and a mutant strain lacking 5′NT activity *L. reuteri* DSM 17938 Δ*sdpA* (Δ5′NT)^35^^,^^41^ were subcultured in LDM4 or MRS media for 24 h. Cell-free supernatant was sampled at 6, 12, and 24 h, and the secreted 5′NT activity was quantified. When grown in MRS, DSM 17938 5′NT activity increased over time during exponential growth (Figure 1A). As expected, activity was absent in the Δ5′NT mutant cultures (Figure 1A, 1B). Surprisingly, the 5′NT activity of *L. reuteri* DSM 17938 strain was lost when cultured in LDM4 medium (Figure 1B).

To check for any growth defects due to the loss of 5′NT activity in the ∆5’NT mutant, cell growth was monitored under both media conditions (Figure 1C, D). As expected, MRS medium supported faster growth of both strains, with observable differences in growth rate. Doubling time of the Δ5′NT mutant was 55 min compared to 40 min for the DSM 17938 strain (Figure 1C Supp. Fig. S1A). A similar growth defect of Δ5′NT mutant was observed in LDM4 medium (doubling time 234 min) compared to 143 min for 17,938 (Figure 1D, Supp. Figure S1B). Because we were unsuccessful in generating a strain to test the complementation of ∆5′ NT for technical reasons, we cannot conclude that the growth defect was due to the loss of 5′NT activity. Taken together, these results suggested that either a component in LDM4 suppressed 5’NT activity or that LDM4 medium lacked the necessary component for 5′NT activity.

### Zinc is required for *L. reuteri’s* 5′ectonucleotidase activity

**Figure 2.**
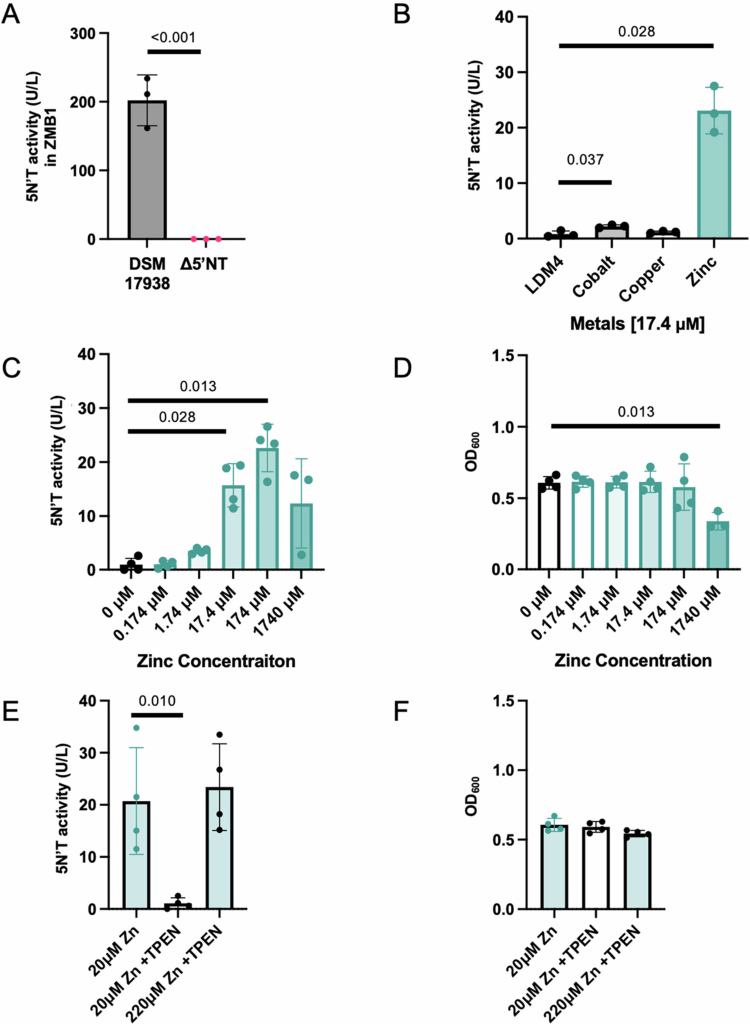
Metal cations differentially impact the 5′ ectonucleotidase activity of *L. reuteri* DSM 17938. *L. reuteri* DSM 17938 and Δ5′NT were grown in ZMB1. Supernatants were collected at 24 h. 5′NT activity was measured in both *L. reuteri* DSM 17938 and Δ5’NT supernatants (A). *L. reuteri* DSM 17938 was grown in LDM4 with the addition of cobalt, copper, or zinc at 17.4 μM for 6 h. 5’NT activity was quantified in supernatants collected from cultures with each metal (B). Next, *L. reuteri* DSM 17938 cultures were grown with increasing concentrations of zinc. The OD_600_ was measured for culture density (D), and 5’NT activity was quantified in the respective supernatants (C). The zinc chelator TPEN was added to cultures with either 20 μM zinc or 220 μM zinc. The 5’NT activity in the supernatants (E) and the OD_600_ of the cultures were measured. Student's *t* test (A) or one-way ANOVA with Dunnett's multiple comparison correction (B–F) was used to determine statistical significance. *N* = 3–4.

To investigate whether nutrient limitation in LDM4 explained the loss of 5′NT activity in LDM4 cultures, we assessed growth and 5′NT activity in a second defined medium, ZMB1. ZMB1 is similar to LDM4 but contains additional metals, vitamins, and fatty acids that support increased cell growth.^37^ Both the DSM 17938 and Δ5′NT strains were cultured for 24 h, and cell-free supernatants were collected. 5′NT activity was restored in DSM 17938, indicating that a factor present in ZMB1 but not LDM4 was required for 5′NT activity (Figure 2A).

Bacterial 5′ectonucleotidases often require metal cation cofactors for activity;^43^ therefore, we sought to determine whether supplementation of LDM4 medium with the additional metal components of ZMB1 medium restored 5′NT activity of the DSM 17938 strain. LDM4 was supplemented with either cobalt, copper, or zinc at 17.4 μM, and 5′NT activity was compared to that of the control medium without added metals. Addition of cobalt supported modest 5′NT activity above control; however, zinc supplementation supported the most robust 5′NT (Figure 2B). Furthermore, zinc supplementation increased 5′NT activity in a dose–dependent manner (Figure 2C). Addition of metals did not significantly alter growth (Supp. Figure S2) until extremely high levels of zinc that resulted in toxicity (Figure 2D). We confirmed these findings using a parallel approach of limiting zinc availability by adding 30 µM zinc chelator N, N, N′ N′-Tetrakis (2-pyridymethyl) ethylenediamine (TPEN) to LDM4 supplemented with either 20 µM or 220 µM zinc. Excess TPEN eliminated 5′NT activity, but oversaturation of TPEN with additional zinc rescued the phenotype (Figure 2E) without altering cell growth (Figure 2F). These data demonstrate that zinc is an essential cofactor for 5′NT activity of *L. reuteri* DSM 17938.

### *L. reuteri* 5′ectonucleotidase activity stimulates melatonin secretion from HIOs

**Figure 3.**
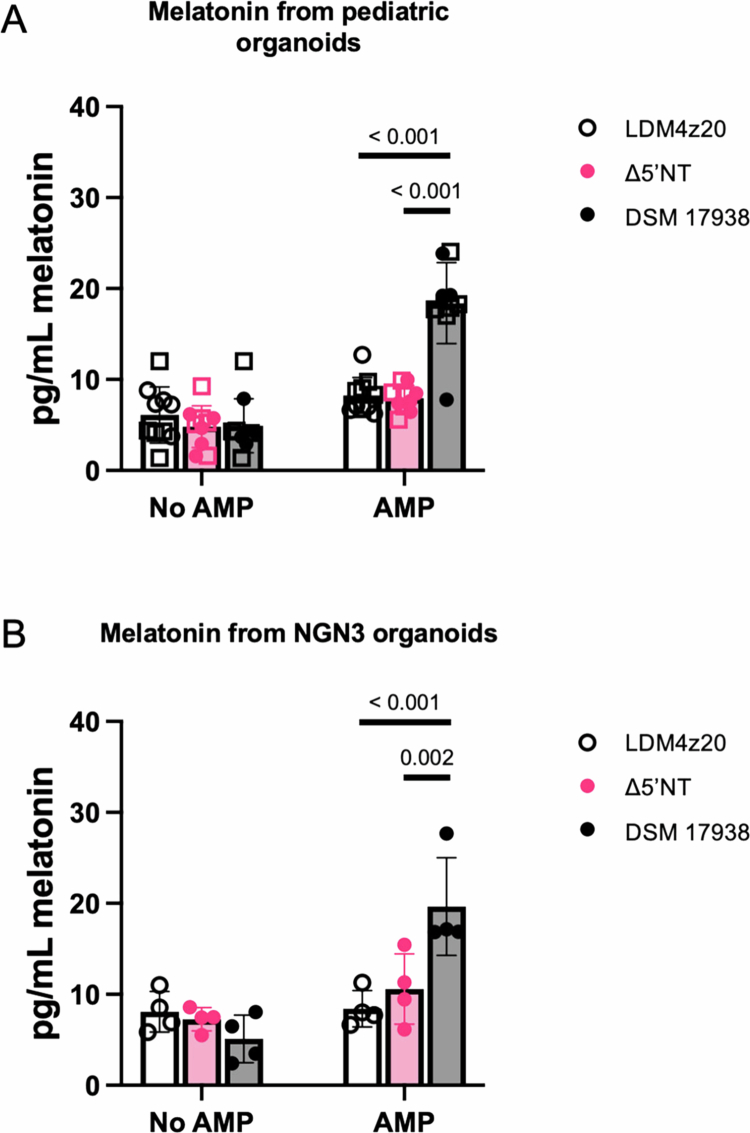
Melatonin release from human intestinal organoids due to 5’ectonucleotidase activity in *L. reuteri* supernatant. Supernatant from *L. reuteri* DSM 17938 and Δ5’NT grown in LDM4 plus 20 μM zinc were incubated with or without AMP for 30 minutes before adding to jejunal pediatric monolayers from two individual donors (A) or the *NGN3* engineered human intestinal organoid monolayer induced for increased numbers of enteroendocrine cells (B). Melatonin in organoid-conditioned media was quantified. *N* = 10 and *N* = 4 biological replicates, respectively for A and B. The shapes in the graphs with pediatric organoids represent two independent infant organoid jejunal lines: ● represents the first pediatric line, and □ represents the second pediatric line. Two-way ANOVA followed by multiple comparisons with Tukey's correction was used to test for statistical significance.

To investigate our hypothesis that *L. reuteri* DSM 17938 5′NT activity promotes the secretion of gut melatonin, we utilized human intestinal organoid (HIO) lines derived from the jejunal epithelium of infants. These two independent pediatric HIO lines were selected based on the finding that they have increased numbers of enteroendocrine cells compared to adult jejunal organoids,^39^ which will facilitate melatonin detection. HIO monolayers were treated for 3 h under the following conditions: LDM4z20, Δ5′NT supernatant, or DSM 17938 supernatant supplemented with adenosine monophosphate (AMP), a known substrate of 5′NT, or vehicle control. *L. reuteri* DSM 17938 supernatant supplemented with AMP significantly promoted melatonin secretion above background levels (Figure 3A), and secretion depended upon both 5’NT activity and AMP (Figure 3A). To determine whether this phenotype was specific to infant organoids, we also tested adult jejunal HIOs engineered to increase enteroendocrine cells by doxycycline-induced overexpression of the transcriptional regulator *NGN3*^40^ under the same conditions as the pediatric organoids. Consistent with the pediatric organoid data, *L. reuteri* DSM 17938 supernatant significantly stimulated melatonin secretion above both LDM4 and Δ5′NT supernatants in an AMP-dependent manner (Figure 3B).

### Carbon source utilization impacts 5′ectonucleotidase activity

Nutrient availability, especially sugars, has broad impacts on the physiology of both the intestinal microbes and the host.^44^ For *L. reuteri* DSM 17938, secreted antimicrobial^45^ products and anti-inflammatory^46^ signals are altered by the media carbohydrate source. Therefore, we investigated whether carbon source utilization impacted *L. reuteri* 5’NT activity. To answer this question, we screened a wide range of physiologically relevant carbon sources simultaneously using Biolog Phenotypic Microarray plates for PM1 and PM2A (Supp. Table S5, full results). Plates were inoculated with *L. reuteri* DSM 17938 cultures containing 200 μM zinc to observe which carbon sources supported growth and 5′NT activity. We observed that the following carbon sources supported growth compared to the no-carbon control: D-galactose, D-gluconic acid, *α*-D-glucose (glucose), maltose, D-melibiose, *α*-D-lactose, lactulose, sucrose, D-raffinose (raffinose), and stachyose (Supp. Figure. S3A, B and Supp. Table S5). Following 10 h of incubation, the supernatant was collected from the plate, and 5′NT activity was quantified (Supp. Figure S3C, D). As expected, bacterial growth (Figure S2A, B) was required for 5′NT activity (Figure S3C, D). Analysis of sugars of interest that are known to modulate *L. reuteri* metabolism and growth^45^^,^^47^ revealed variance in 5′NT activity. Interestingly, stachyose and raffinose had the highest levels of 5′NT activity, even under poor growth conditions (Figure S3 E, F). These findings suggest that carbon source utilization directly impacts 5′NT activity.

**Figure 4.**
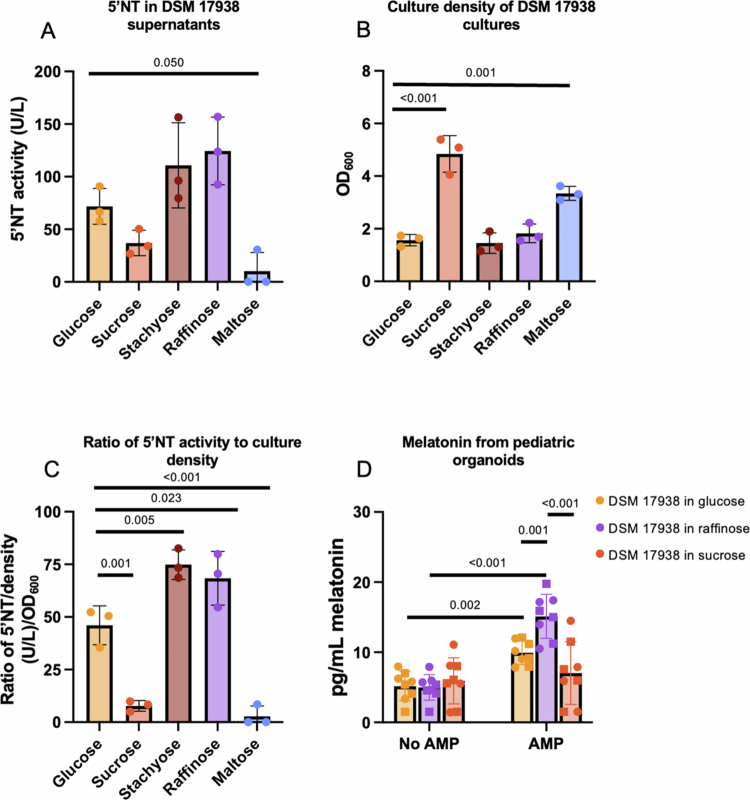
Carbon source impact on 5′ectonucleotidase activity and subsequent stimulation of melatonin release from pediatric intestinal organoids. *L. reuteri* DSM 17938 was grown in LDM4 with 20 mg/mL various carbon sources supplemented with 200 μM zinc for 24 h. 5’NT activity in the supernatant was quantified (A), culture density was measured at OD_600_ (B), and the ratio of 5′NT activity to culture density was calculated (C). The ratio of 5’NT activity to culture density was calculated by dividing the raw 5′NT activity (U/L) in the supernatant by the culture growth (OD_600_). *N* = 3 biological replicates. Separately, *L. reuteri* DSM 17938 supernatant was collected from cultures grown in LDM4 with glucose, raffinose, or sucrose as the carbon source supplemented with 200 μM zinc. Undiluted supernatants collected from cultures with different carbon sources were combined with or without AMP and immediately added to the two pediatric jejunal lines, and melatonin in organoid-conditioned media was quantified (D). *N* = 8 biological replicates. The shapes of the graphs with pediatric organoids represent two independent infant organoid jejunal lines: ● represents the first pediatric line, and □ represents the second pediatric line. One-way ANOVA with Dunnett’s multiple comparison correction was used to determine statistical significance for (A), (B), and (C). Two-way ANOVA followed by multiple comparisons with Tukey's correction was used to test for statistical significance for (D).

Because the concentrations of carbon sources in Biolog plates are proprietary, we further investigated a subset of these carbon sources under fully defined experimental conditions. *L. reuteri* DSM 17938 was cultured in LDM4z200 with 20 mg/mL glucose, sucrose, stachyose, raffinose, or maltose. After 24 h of incubation, the supernatant was collected for 5′NT analysis (Figure 4A), and OD_600_ was measured (Figure 4B). Compared to control cultures containing glucose, only the maltose cultures presented significantly lower 5′NT activity, and 5′NT activity in the cultures containing stachyose and raffinose tended to be 50% higher than that in the glucose cultures (Figure 4A). As we observed in our screen, the culture densities varied among the carbon sources (Figure 4B). These growth results align with prior work highlighting *L. reuteri’s* preference for growth on sucrose and maltose.^47^ When 5′NT activity is normalized to the culture density, cultures grown in stachyose and raffinose media have significantly increased ratios of 5′NT to culture density compared to cultures grown in glucose (Figure 4C). Conversely, cultures grown with either sucrose or maltose have significantly lower ratios of 5′NT activity to culture density as compared to cultures grown in glucose (Figure 4C).

### *L. reuteri* 5′ectonucleotidase activity correlates with melatonin release from HIOs

To further evaluate the impact of variation in 5′NT activity on epithelial melatonin stimulation, we treated pediatric HIO monolayers with supernatants collected from *L. reuteri* DSM 17938 cultured in LDM4z200 supplemented with 20 mg/mL glucose, raffinose, or sucrose for 24 h. To reduce background 5’NT activity and better distinguish differences, we eliminated the preincubation of supernatants with AMP or the vehicle control prior to the melatonin secretion assay. In conditions with AMP, raffinose cultures stimulated more melatonin release than did cultures with glucose or sucrose (Figure 4D). Consistent with the reduced 5′NT activity of the cells grown in sucrose, the supernatant from the sucrose cultures did not stimulate melatonin release above the control conditions lacking AMP (Figure 4D). Collectively, these data support the role of *L. reuteri* 5′NT in promoting the secretion of melatonin from intestinal epithelial cells.

### Melatonin release is dependent upon adenosine and a second *L. reuteri* secreted factor

Following our observation that 5′NT activity and AMP are required for *L. reuteri*-driven release of melatonin, we tested whether exogenous adenosine or inosine, a metabolite derivative of adenosine which *L. reuteri* also elevates in mice,^28^ was sufficient to promote melatonin release. LDM4, LDM4 with adenosine, and LDM4z200 with inosine were added to pediatric organoids to test for melatonin secretion. Unexpectedly, both the exogenous adenosine and inosine were insufficient to stimulate melatonin release from the organoids (Supp. Figure 4).

**Figure 5.**
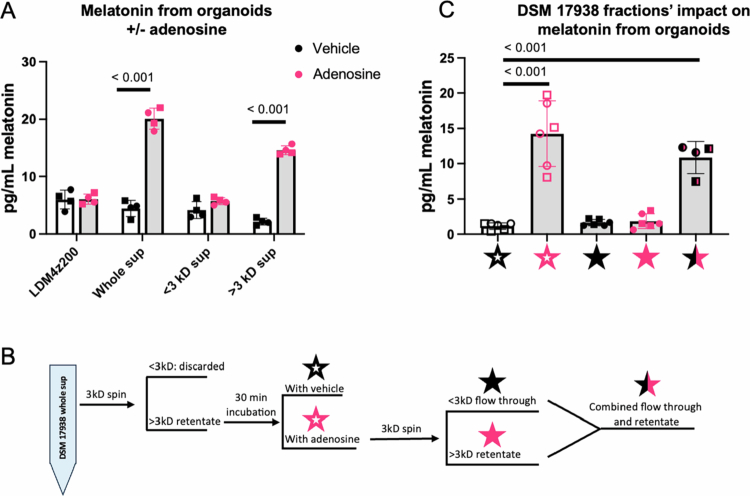
An unknown factor in *L. reuteri* DSM 17938 supernatant in combination with adenosine is required for melatonin release from organoids. Media control and different fractions of *L. reuteri* DSM 17938 supernatant (whole, <3 kD, and >3 kD) incubated with (pink) or without (black) 2 mM adenosine were added to the pediatric organoids, and melatonin was measured (A). The pipeline for generating the supernatant samples utilized in (C) is shown in (B). Each star’s color corresponds to its respective condition in (C). In brief, a >3 kD fraction of *L. reuteri* DSM 17938 was incubated with or without adenosine for 30 min. The fraction receiving adenosine was then size fractionated through a 3 kD filter once more. Finally, aliquots from the resulting size fractionation were recombined. In graphs (A) and (C), points shaped like ● represent the first pediatric line, and points shaped as □ represent the second pediatric line. *N* = 4–6. Multiple unpaired *t* tests with two-stage step-up (Benjamini, Krieger, and Yekutieli) corrections were performed for (A). One-way ANOVA followed by Dunnett's correction for multiple comparisons was performed for (C).

After observing that neither adenosine nor inosine alone was sufficient to stimulate melatonin release, we wanted to determine whether the addition of adenosine to *L. reuteri* supernatant or a particular size fractionation of *L. reuteri* supernatants could promote melatonin secretion from organoids. Supernatants from 24-h *L. reuteri* DSM 17938 cultures grown in LDM4z200 were fractionated by size exclusion centrifugal filters. The resulting <3 kD fraction, >3 kD fraction, and whole supernatant were incubated with or without adenosine prior to the 1-h treatment of the pediatric HIO monolayers. Addition of adenosine to the whole supernatant resulted in a significant increase in melatonin, as anticipated. The addition of adenosine to the LDM4z200 control did not increase melatonin levels (Figure 5A). Furthermore, the addition of adenosine to the >3 kD fraction, but not the <3 kD fraction, was also able to significantly increase melatonin levels (Figure 5A).

To further investigate these findings, we next set up a pipeline of *L. reuteri* DSM 17938 supernatant processing to further assess other enzymatic reactions or secondary secreted factors promoting melatonin release (Figure 5B). In brief, the >3 kD fraction of *L. reuteri* supernatant was incubated with or without adenosine, and then, the >3 kD fraction incubated with adenosine was fractionated again through a 3 kD filter. The resulting flow through, retentate, and combined mixture of flow through and retentate were collected (Figure 5B). The color of the star in Figure 5B corresponds to the color of symbols highlighting samples in Figure 5C. Consistent with the previous experiment, incubating the >3 kD *L. reuteri* fraction with adenosine resulted in melatonin-stimulating activity (Figure 5C). However, when this mixture was fractionated a second time, melatonin-stimulating activity was lost in each of the resulting fractions. Because of adenosine's low molecular weight, we would expect adenosine and any secondary adenosine metabolites synthesized by *L. reuteri* to be in this second <3 kD fraction. Therefore, we conclude that adenosine is not converted to another small signaling molecule sufficient to stimulate melatonin release from pediatric HIOs. Similarly, the second >3 kD fraction lost activity. Because recombining these fractions significantly stimulated melatonin release from the pediatric organoids (Figure 5C), we conclude that there are two independent factors required for melatonin secretion.

### Industrial preparation of *L. reuteri* BG-R46^®^ stimulates melatonin release from HIOs

**Figure 6.**
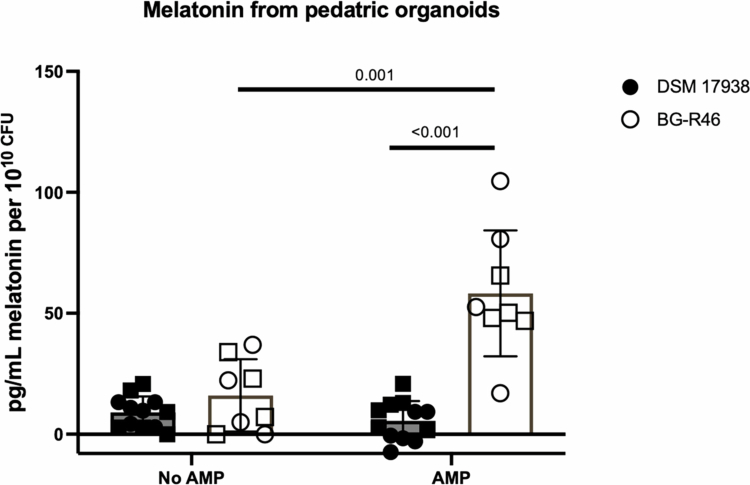
Melatonin release from human intestinal organoids in response to *L. reuteri* strains DSM 17938 and BG-R46^®^. Supernatants from commercially produced batches of *L. reuteri* DSM 17938 and BG-R46^®^ were incubated with or without AMP for 30 min before adding to jejunal pediatric monolayers from two individual donors. Melatonin in organoid-conditioned media was quantified. The shapes of the graphs with pediatric organoids represent two independent infant organoid jejunal lines: ● represents the first pediatric line, and □ represents the second pediatric line. Values were normalized against a lyoprotectant control and normalized to pg/mL per 10^10^ CFU. N_DSM 17938_ = 12 and N_BG-R46®_ = 8. Two-way ANOVA followed by multiple comparisons with Fisher's LSD was used to test for statistical significance.

The process of generating *L. reuteri* for human supplementation differs from standard laboratory growth processes, thereby presenting a possible point of divergence in terms of metabolic output impacting HIO melatonin release. To validate whether these commercially made cultures could stimulate melatonin release, industrially produced batches of *L. reuteri* DSM 17938 and *L. reuteri* BG-R46^®^, a novel strain derived from DSM 17938 known to have increased 5′NT activity over 17938,^32^^,^^41^ supernatants were obtained from BioGaia. These supernatants and the lyoprotectant control were combined with AMP or vehicle control prior to addition to pediatric HIOs. While the commercially generated *L. reuteri* DSM 17938 supernatant with AMP did not significantly increase melatonin release, the *L. reuteri* BG-R46^®^ batch did significantly increase melatonin secretion when AMP was added (Figure 6). Consistent with previous work showing *L. reuteri* BG-R46^®^ (DSM 32846) having increased 5’NT to greater than *L. reuteri* DSM 17938,^32^ a more than tenfold increase in melatonin secretion upon AMP addition was observed compared to *L. reuteri* DSM 17938 (Figure 6). The strains did not stimulate melatonin under conditions when no AMP was added.

## Discussion

**Figure 7.**
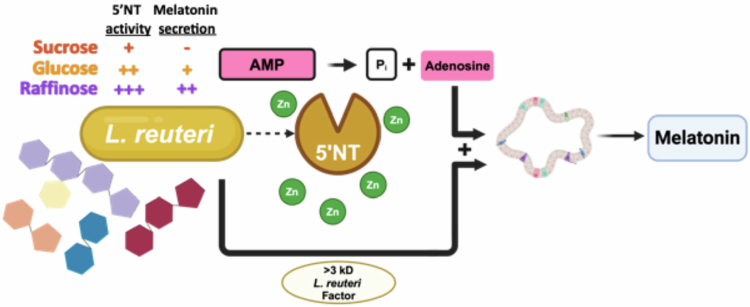
*L. reuteri* promotes the secretion of melatonin from HIOs through 5′NT activity and another *L. reuteri* factor. Working model for *L. reuteri* mediated the secretion of gut melatonin. An *L. reuteri* >3 kD factor- and 5′NT-generated adenosine are required simultaneously to stimulate the release of melatonin from HIOs. Zinc is an essential cofactor of 5′NT activity. The carbon source upon which *L. reuteri* is grown alters 5′NT levels, and corresponding melatonin release correlates to respective 5’NT levels.

Understanding how orally ingested bacteria impact host health and physiology is critical for developing future probiotic and live biotherapeutic applications. Numerous clinical trials and meta-analyses^5^^,^^6^^,^^48^^,^^49^ have shown that *L. reuteri* DSM 17938 is effective in ameliorating infant colic symptoms, though mechanisms are poorly understood. We focused on the link among *L. reuteri*, adenosine signaling, and gut melatonin secretion as a possible mechanism for multiple reasons. First, melatonin is produced in the gut^19^ and has been proposed to be involved in colic etiology.^11^^,^^12^ Second, both melatonin^17^ and *L. reuteri*^2^ are known to alleviate abdominal pain. Third, adenosine signaling is known to regulate the secretion of brain melatonin.^24^^,^^25^ Fourth, *L. reuteri* DSM 17938 increases survival rates in a lethal autoimmune disorder in mice dependent on the adenosine 2A receptor.^29^ Finally, *L. reuteri* DSM 17938 is able to generate extracellular adenosine through its 5′NT enzyme.^32^ Taken together, these observations led us to the experiments tested in this study, resulting in the findings illustrated by the model in Figure 7.

In this work, we observed *L. reuteri* stimulation of melatonin release from human intestinal epithelial organoids required 5′NT activity and the presence of AMP. Interestingly, commercially produced BG-R46, a strain demonstrated to exhibit increased secreted 5′NT activity,^32^^,^^41^ was also shown to be more potent than commercially produced DSM 17938. We speculate that the lack of a potent response from the commercially produced DSM 17938 is due to the differences in growth and processing parameters between laboratory-generated supernatants and commercially prepared lyophilized cultures. Furthermore, *L. reuteri* BG-R46 is known to have increased amounts of 5′NT activity compared to *L. reuteri* DSM 17938 even in commercial preparations.^34^ We utilized pediatric organoids in part because of their physiological relevance for infantile colic. Interestingly, the pediatric organoid lines we used in this study contained a higher number of enteroendocrine cells than adult organoids.^39^ The pediatric HIO platform allowed us to investigate *L. reuteri's* impact on melatonin production and allowed partial insight into the molecular basis of melatonin stimulation. The precise mechanism by which *L. reuteri* stimulates melatonin release, and how this may impact symptoms of colic remains to be elucidated.

Surprisingly, adenosine was necessary but not sufficient to stimulate melatonin release. By conducting size fractionation of *L. reuteri* DSM 17938 supernatants, we determined that the addition of adenosine to the >3 kD fraction was sufficient to stimulate melatonin release. We suspected that the additional enzymatic activity harbored in the >3 kD could be converting adenosine into another small molecule that stimulates melatonin secretion. This possibility was excluded when the >3 kD fraction containing adenosine was secondarily size fractionated, and the resulting <3 kD fraction, which would contain any adenosine metabolites, did not stimulate melatonin release. However, the addition of both the <3 kD and >3 kD secondary fractions, each inactive by themselves, stimulated melatonin secretion. This strongly suggests that both adenosine (or a metabolite of adenosine) and an unidentified *L. reuteri* factor are simultaneously required to stimulate intestinal melatonin secretion. BG-R46^®^ can also stimulate melatonin release when AMP is added, and the secondary factor in this strain is likely the same between the two strains, as BG-R46^®^ is a direct descendant of DSM 17938.

The physiological relevance of key molecule concentrations, particularly AMP and melatonin, is important to consider for the broader context of this work. Extracellular purinergic signaling, including ATP and AMP levels, is a response to cellular damage and inflammation. Intracellular concentrations of ATP can be in the single-millimolar range.^50^ If lysed due to damage or severe inflammation, this would result in very high local concentrations of ATP and thereby AMP due to the catalytic activity of ectoenzyme CD39.^51^ This is further corroborated by the evidence showing ATP receptor P2X7, an important receptor involved in intestinal inflammation^52^ is not fully activated until ATP reaches the millimolar range.^53^ Therefore, our pg/mL of AMP is within physiologically relevant concentrations within the human intestine. There are two GPCR receptors for melatonin, MT1 and MT2. MT1 has a Ki value of approximately20 pg/mL, and MT2 has a Ki value of approximately 90 pg/mL.^54^ The concentration of melatonin measured from organoids is near the effective range of MT1. Furthermore, nighttime serum levels of melatonin change based on age. Nighttime melatonin concentrations are low before 6 months of age, at approximately 27 pg/mL. However, nighttime levels thereafter quickly increase to approximately 330 pg/mL in the first few years of life before tapering off back to near 30 pg/mL.^55^ Our pediatric organoid release of melatonin is near the nighttime concentration of serum melatonin for infants under 6 months of age.

Given that carbon substrates are known to alter *L. reuteri* enzymatic outputs,^45^^,^^46^ we tested and observed that carbon sources modify the 5′NT activity levels of *L. reuteri*. It remains to be determined whether the expression of the 5′NT gene *sdpA* is under direct carbon catabolism regulation or whether more global expression regulation due to the carbon source is occurring. One potential candidate involved in 5′NT activity is *scrR*, an important transcriptional regulator of sucrose metabolism genes.^56^ Another possible explanation for the difference in 5′NT activity could be a difference in extracellular membrane vesicle secretion given that 5′NT is present and active on the surface of extracellular membrane vesicles.^32^ This raises the possibility that *L. reuteri* grown on different carbohydrate sources could alter the number of extracellular membrane vesicles released. Further work is needed to elucidate whether the increase in *L. reuteri* 5′NT activity when grown on stachyose and raffinose is due to increased expression or increased extracellular membrane vesicles.

Demonstrating an essential role of zinc and carbon sources in modulating *L. reuteri* 5′NT activity levels support the significant role diet may play in melatonin secretion in the gastrointestinal tract. Having shown the amount of 5′NT activity from cells grown in different carbon sources correlates with the amount of secreted melatonin from pediatric HIOs, the question arises of whether these findings translate to *in vivo* or clinical models. The impact that supplemental carbon sources and zinc could have on patient outcomes should be considered and experimentally investigated while developing *L. reuteri* 5′NT-based biotherapeutic products. Uncovering these mechanisms provides future lines of investigation for researchers and clinicians to better understand the interactions of *L. reuteri* and host physiology with the aim of developing more effective *L. reuteri* treatments for patient conditions such as infant colic. Owing to its increased induction of melatonin release, BG-R46^®^ is a candidate for improving the treatment and prevention of infant colic. Interestingly, an ongoing clinical study is investigating the therapeutic potential of combining DSM 17938 and BG-R46^®^ to alleviate symptoms and develop a more effective treatment of infant colic.

## Supplementary Material

Supplementary Material Supplementary Material

## Data Availability

The data that support the findings of this study are openly available at figshare. doi: 10.6084/m9.figshare.30524492.

## Abbreviations

5’NT: (5’ectonucleotidase)
HIO: (human intestinal organoid)
AMP: (adenosine monophosphate)
