# Supplementary material for: Limosilactobacillus reuteri promotes melatonin release from human intestinal organoids via 5′ectonucleotidase activity
Source: Gut Microbes. 2026 May 18;18(1):2670854. doi: 10.1080/19490976.2026.2670854 (PMC13192094; doi:10.1080/19490976.2026.2670854)
Supplement: Supplementary Material [file KGMI_A_2670854_SM6367.docx]

**Supplemental Figure S1. *L. reuteri* doubling times in MRS and LDM4.**

*L. reuteri* DSM 17938 and Δ5’NT were grown for 24 hours in MRS and LDM4. Doubling times of exponential growth for both strains were calculated for growth in MRS (A) and in LDM4 (B). A student t test was used to determine statistical significance between doubling times of *L. reuteri* DSM 17938 and Δ5’NT strains. N = 4 biological replicates.


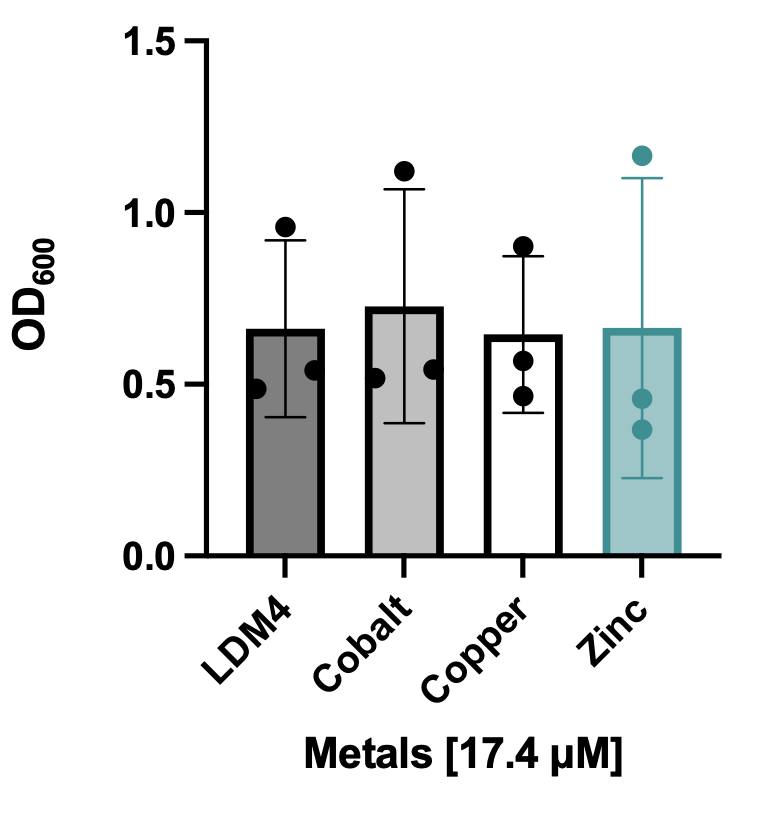


**Supplemental Figure S2. *L. reuteri* DSM 17938 growth in LDM4 with different metals.**

*L. reuteri* DSM 17938 was grown in LDM4 with either cobalt, copper, or zinc at 17.4 μM for six hours. OD_600_ was measured after six hours of 37°C incubation. A one-way ANOVA with Dunnett’s multiple comparison corrections was used to test for statistical significance. N = 3.


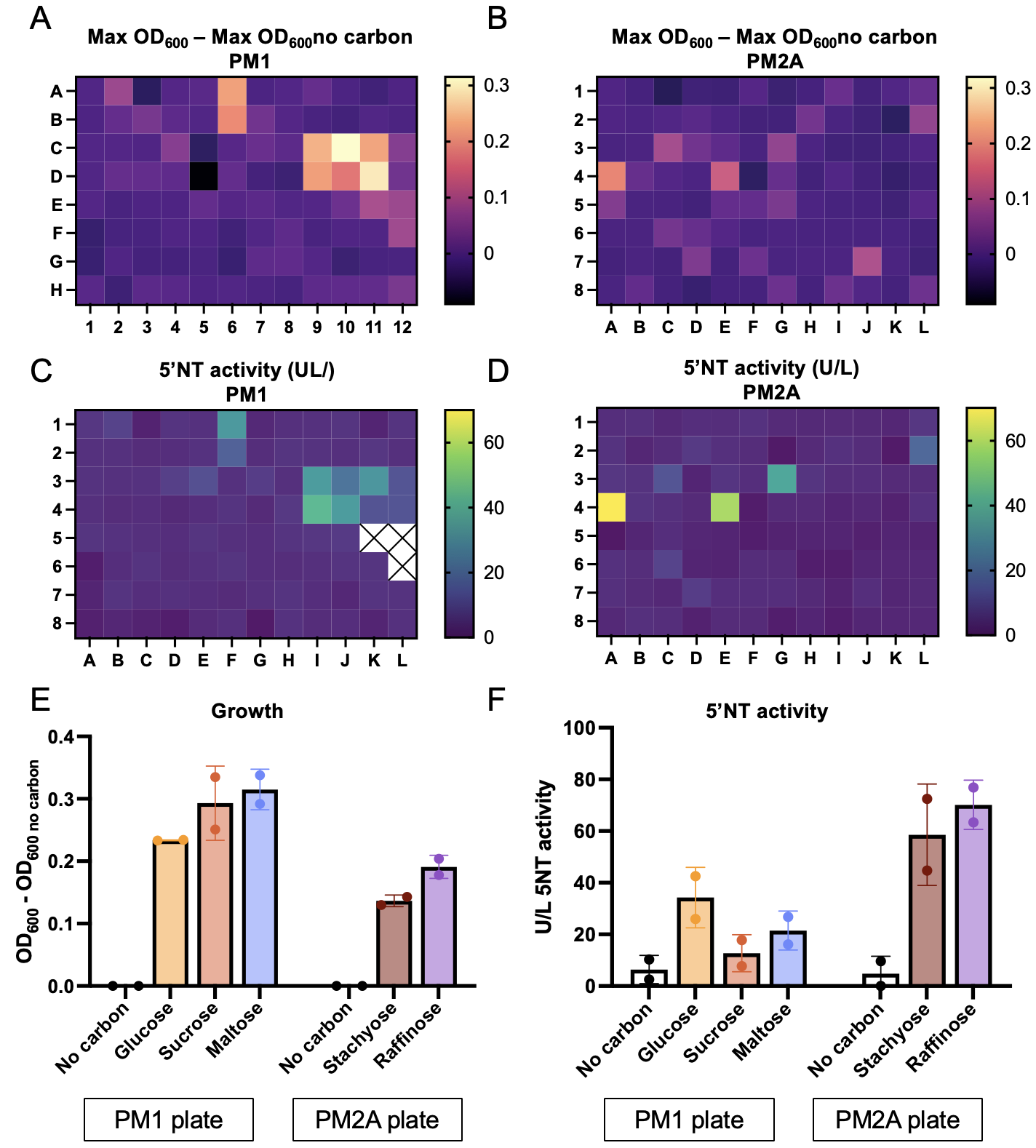


**Supplemental Figure S3. Growth and 5’ectonucleotidase activity of *L. reuteri* on carbon sources of Biolog plates.** *L. reuteri* DSM 17938 was grown on Biolog plates PM1 and PM2A in glucose-free LDM4 with 200 μM zinc. Growth was calculated by subtracting the max OD_600_ of the no carbon control (well A1) in either PM1 (A) or PM2A (B) plates from the max OD_600_ of each respective well (A, B). 5’NT activity from of each Biolog plate was quantified (C, D). Boxes with “X” contained substrates that interfered with the assay quantifying 5’NT activity. Specific carbon sources of interest had its growth (E) and 5’NT activity (F) plotted individually. N = 2.

**
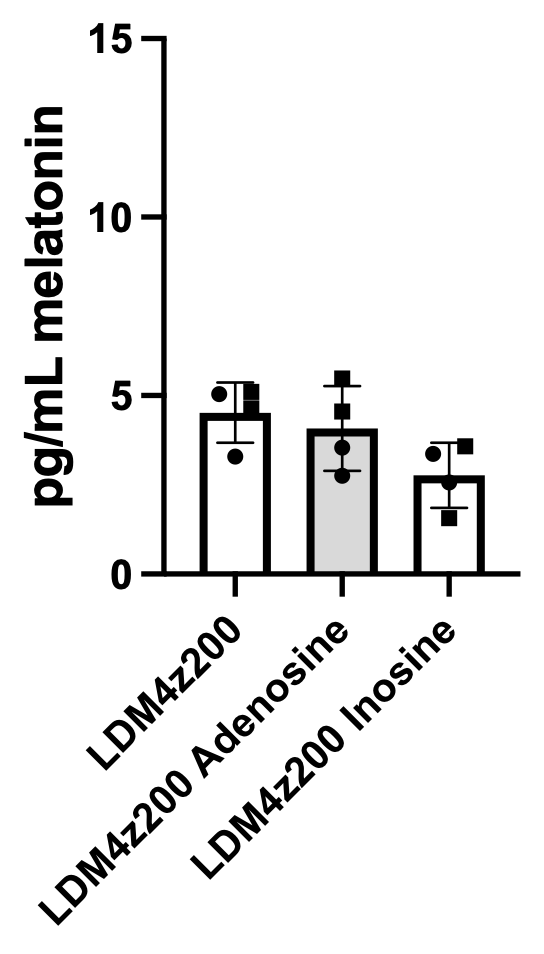
**

**Supplemental Figure S4. Inosine does not stimulate release of melatonin from intestinal organoids alone.**

LDM4 alone, LDM4 with 2 mM adenosine, and LDM4 with 2 mM inosine was added to pediatric HIO monolayers. The resulting organoid conditioned media was quantified for melatonin. A one-way ANOVA with Dunnett’s multiple comparison corrections was used to determine statistical significance. Points shaped like ● represent the first pediatric line, and points shaped as ◼︎ represent the second pediatric line. N = 4.

Supplemental Table S1. Bacterial Strains used in this study

| **Bacteria** | **Characteristics^†^** | **Source** |
| --- | --- | --- |
| *L. reuteri* DSM 17938 | Plasmid-cured derivative of *L. reuteri* ATCC 55730 | BioGaia  (33) |
| *L. reuteri* DSM 17938 Δ*sdpA*  (Δ5’NT) | In-frame deletion of 2.2 kb *sdpA* in *L. reuteri* DSM 17938 | (35) |
| *L. reuteri* BG-R46^®^ | An evolved strained derived from DSM 17938 | BioGaia  (34) |

**^†^** *sdpA*: bifunctional metallophosphatase/5’-nucleotidase (NCBI Accession# WP_003672341.1)

Supplemental Table S2. Recipes for individual components of LDM4

| **Solution** | **Ingredient** | **Catalog#** | **Amount** |
| --- | --- | --- | --- |
| **10x Basal Medium** | K_2_HPO_4_ * 3 H_2_O | Sigma P5504 | 6.5 g |
|  | KH_2_PO_4_ Potassium Phosphate | Sigma P5655 | 25 g |
|  | Sodium Acetate | Sigma S5636 | 5 g |
|  | Ammonium Citrate Tribasic | Sigma A1332 | 4 g |
|  | L- Ascorbic Acid | Sigma 255564 | 2.5 g |
|  | Sodium Chloride | Sigma S5886 | 6 g |
|  | DI water |  | 500 mL |
|  |  |  |  |
| **Vitamin Solution** | Thiamin hydrochloride (Thiamine) | Sigma T3902 | 0.01 g |
|  | 4-aminobenzoic acid | Sigma A9878 | 0.002 g |
|  | D-Panthothenic acid hemicalcium salt | Sigma P2250 | 0.02 g |
|  | Niacin (Nicotinic acid) | Sigma N0761 | 0.05 g |
|  | Pyridoxine hydrochloride | Sigma P6280 | 0.025 g |
|  | DI water |  | 25 mL |
|  |  |  |  |
| **Biotin Solution** | Biotin | Sigma B4501 | 0.005 g |
|  | 95 % Ethanol | Sigma E702-3 | Few drops |
|  | HCl, 0.01 M |  | 50 ml |
|  |  |  |  |
| **Riboflavin Solution** | Riboflavin | Sigma R9504 | 0.004g |
|  | Acetic Acid 0.02 M | Sigma 27221 [99-100%] | 50 ml |
|  |  |  |  |
| **Folic Acid** | Folic Acid | Sigma F8758 | 0.01 g |
|  | NaOH, 0.01 M | Sigma 8045 | 50 ml |
|  |  |  |  |
| **Nucleic Acid Solution** | Adenine sulfate | Sigma A3159 | 0.05 g |
|  | Guanine hydrochloride | USB 16740 | 0.05 g |
|  | Cytidine 5’-monohosphate (4°C) | Sigma C1131 | 0.1 g |
|  | HCl, 1 M | Sigma H9892 | 15 ml |
|  |  |  |  |
| **Uracil Solution** | Uracil | Sigma U1128 | 0.2 g |
|  | NaOH, 1 M | S8045 | 10 ml |
|  |  |  |  |
| **Thymidine Solution** | Thymidine | Sigma T1895 | 0.02 g |
|  | DI Water |  | 12.5 ml |
|  |  |  |  |
| **Salt Solution** | MgSO_4_ x 7 H_2_O | Sigma M1880 | 1.625 g |
|  | MnSO_4_ x H_2_O | Sigma M7899 | 0.143 g |
|  | FeSO_4_ x 7 H_2_O | Sigma F8048 | 0.130 g |
|  | MilliQ Water |  | 10 mL |
|  |  |  |  |
| **Glucose Solution** | Glucose | Sigma G8270 | 200 g |
|  | MilliQ Water |  | 500 ml |
|  |  |  |  |
| **Amino Acid Solution** | L- Alanine | Sigma A7469 | 0.24 g |
|  | L-Arginine | Sigma A8094 | 0.125 g |
|  | L-Asparagine monohydrate | Sigma A4284 | 0.2 g |
|  | L-Aspartic Acid | Sigma A8949 | 0.42 g |
|  | L-Cysteine hydrochloride monohydrate | Alfa Aesar A10389-22 | 0.2 g |
|  | L-Glutamic Acid | Fluka 49450 | 0.5 g |
|  | Glycine | Sigma G8790 | 0.175 g |
|  | L-Histidine | Sigma H8000 | 0.15 g |
|  | L-Isoleucine | Sigma I7403 | 0.21 g |
|  | L-Leucine | Sigma L8912 | 0.475 g |
|  | L-Lysine monohydrochloride | Sigma L8662 | 0.55 g |
|  | L-Methionine | Sigma M5308 | 0.125 g |
|  | L-Phenylalanine | Sigma P5482 | 0.275 g |
|  | L-Proline | Sigma P5607 | 0.675 g |
|  | L-Serine | Sigma S4311 | 0.34 g |
|  | L-Threonine | Sigma T8441 | 0.225 g |
|  | L-Tryptophane | Sigma T8941 | 0.05 g |
|  | L-Tyrosine | Sigma T8566 | 0.25 g |
|  | L-Valine | Sigma V0513 | 0.335 g |
|  | DI Water |  | 837.5 mL |

Supplemental Table S3. Composition of LDM4

| **Solution** | **Volume (mL)** |
| --- | --- |
| 10x Basal Medium: | 100 |
| Vitamin Solution: | 0.5 |
| Biotin Solution: | 0.5 |
| Riboflavin Solution: | 5 |
| Folic Acid Solution: | 0.5 |
| Nucleic Acid Solution: | 3 |
| Uracil Solution: | 1 |
| Thymidine Solution: | 1 |
| Salt Solution: | 1 |
| Glucose Solution: | 50 |
| Amino Acid Solution: | 837.5 |
| **TOTAL:** | 1000**^†^** |

**^†^**Final solution adjusted to pH 6.5

Supplemental Table S4. Composition of differentiation medium

| **Reagent** | **Brand** | **Stock Concentration, Final concentration** | **Volume (mL)** |
| --- | --- | --- | --- |
| Advanced DMEM/f12 | Gibco ThermoFisher 12634010 | N/A | 8 |
| R-spondin conditioned media | Sigma-Aldrich SCC111 | N/A | 1 |
| Noggin conditioned media | (obtained cell line from Dr. Muncan V. Van den Brink GR)^57^ | N/A | 0.5 |
| N2 supplement | ThermoFisher -17502-048 | 100x, 1X | 0.1 |
| B27 supplement | ThermoFisher 17504-044 | 50X, 1X | 0.2 |
| N-acetylcysteine | Sigma-Aldrich A9165-5G | 500mM, 1 mM | 0.02 |
| Mouse recombinant EGF | ThermoFisher PMG8043 | 50 ug/mL, 50 ng/mL | 0.01 |
| A-83-01 | Torcis 2939 | 500 uM, 500nM | 0.01 |
| [Leu15]- Gastrin I | Sigma-Aldrich G9145 | 10 uM, 10 nM | 0.01 |

Supplemental Table S5. Growth (MaxOD_600_-MaxOD_600_no carbon) and 5’NT activity (U/L) of *L. reuteri* DSM 17938 for every carbon source on each Biolog plates.

| Biolog plate | Carbon Substrate | U/L 5’NT rep 1 | U/L 5’NT rep 2 | Max OD rep 1 | Max OD - MaxOD no carbon rep 1 | Max OD rep 2 | Max OD - MaxOD no carbon rep 2 |
| --- | --- | --- | --- | --- | --- | --- | --- |
| PM1 | Negative Control | 2.540 | 0.000 | 0.277 | 0.000 | 0.300 | 0.000 |
| PM1 | L-Arabinose | 4.532 | 2.098 | 0.351 | 0.074 | 0.383 | 0.083 |
| PM1 | N-Acetyl-D-Glucosamine | 1.161 | 0.000 | 0.237 | -0.040 | 0.274 | -0.026 |
| PM1 | D-Saccharic Acid | 2.976 | 0.834 | 0.278 | 0.001 | 0.304 | 0.004 |
| PM1 | Succinic Acid | 2.744 | 0.851 | 0.277 | 0.000 | 0.313 | 0.013 |
| PM1 | D-Galactose | 20.712 | 1.290 | 0.470 | 0.193 | 0.536 | 0.236 |
| PM1 | L-Aspartic Acid | 1.177 | 0.711 | 0.274 | -0.003 | 0.294 | -0.006 |
| PM1 | L-Proline | 1.608 | 0.176 | 0.274 | -0.003 | 0.305 | 0.005 |
| PM1 | D-Alanine | 1.846 | 0.228 | 0.268 | -0.009 | 0.348 | 0.048 |
| PM1 | D-Trehalose | 2.075 | 0.465 | 0.274 | -0.003 | 0.294 | -0.006 |
| PM1 | D-Mannose | 0.965 | 0.000 | 0.262 | -0.015 | 0.285 | -0.015 |
| PM1 | Dulcitol | 1.749 | 0.000 | 0.275 | -0.002 | 0.296 | -0.004 |
| PM1 | D-Serine | 1.997 | 2.985 | 0.279 | 0.002 | 0.290 | -0.010 |
| PM1 | D-Sorbitol | 2.593 | 0.000 | 0.280 | 0.003 | 0.334 | 0.034 |
| PM1 | Glycerol | 2.442 | 0.000 | 0.283 | 0.006 | 0.371 | 0.071 |
| PM1 | L-Fucose | 2.077 | 5.197 | 0.289 | 0.012 | 0.311 | 0.011 |
| PM1 | D-Glucuronic Acid | 1.819 | 1.826 | 0.279 | 0.002 | 0.298 | -0.002 |
| PM1 | D-Gluconic Acid | 13.080 | 0.000 | 0.514 | 0.237 | 0.449 | 0.149 |
| PM1 | D,L-alpha-Glycerol Phosphate | 0.173 | 0.000 | 0.313 | 0.036 | 0.328 | 0.028 |
| PM1 | D-Xylose | 2.163 | 0.000 | 0.286 | 0.009 | 0.296 | -0.004 |
| PM1 | L-Lactic Acid | 2.075 | 0.799 | 0.281 | 0.004 | 0.280 | -0.020 |
| PM1 | Formic Acid | 1.952 | 0.729 | 0.271 | -0.006 | 0.279 | -0.021 |
| PM1 | D-Mannitol | 2.462 | 0.000 | 0.278 | 0.001 | 0.299 | -0.001 |
| PM1 | L-Glutamic Acid | 2.013 | 5.780 | 0.274 | -0.003 | 0.292 | -0.008 |
| PM1 | D-Glucose-6-Phospate | 1.873 | 2.651 | 0.287 | 0.010 | 0.292 | -0.008 |
| PM1 | D-Galactonic Acid-gamma-Lactone | 2.050 | 1.563 | 0.277 | 0.000 | 0.303 | 0.003 |
| PM1 | D,L-Malic Acid | 2.789 | 9.893 | 0.278 | 0.001 | 0.295 | -0.005 |
| PM1 | D-Ribose | 3.998 | 0.000 | 0.331 | 0.054 | 0.357 | 0.057 |
| PM1 | Tween 20 | 9.238 | 0.720 | 0.254 | -0.023 | 0.259 | -0.041 |
| PM1 | L-Rhamnose | 2.424 | 0.000 | 0.276 | -0.001 | 0.309 | 0.009 |
| PM1 | D-Fructose | 2.824 | 24.895 | 0.304 | 0.027 | 0.317 | 0.017 |
| PM1 | Acetic Acid | 1.925 | 1.887 | 0.289 | 0.012 | 0.308 | 0.008 |
| PM1 | alpha-D-Glucose | 25.987 | 0.421 | 0.511 | 0.234 | 0.533 | 0.233 |
| PM1 | Maltose | 16.132 | 0.614 | 0.615 | 0.338 | 0.592 | 0.292 |
| PM1 | D-Melibiose | 13.481 | 0.000 | 0.470 | 0.193 | 0.546 | 0.246 |
| PM1 | Thymidine | 8.082 | 0.000 | 0.347 | 0.070 | 0.337 | 0.037 |
| PM1 | L-Asparagine | 4.114 | 63.406 | 0.281 | 0.004 | 0.294 | -0.006 |
| PM1 | D-Aspartic Acid | 1.988 | 1.203 | 0.282 | 0.005 | 0.334 | 0.034 |
| PM1 | D-Glucosaminic Acid | 1.917 | 0.009 | 0.288 | 0.011 | 0.324 | 0.024 |
| PM1 | 1,2-Propanediol | 2.050 | 0.000 | 0.278 | 0.001 | 0.327 | 0.027 |
| PM1 | Tween 40 | 2.282 | 44.699 | 0.226 | -0.051 | 0.180 | -0.120 |
| PM1 | alpha-Keto-Glutaric Acid | 2.077 | 0.000 | 0.286 | 0.009 | 0.324 | 0.024 |
| PM1 | alpha-Keto-Butyric Acid | 0.639 | 0.000 | 0.267 | -0.010 | 0.287 | -0.013 |
| PM1 | alpha-Methyl-D-Galactoside | 2.110 | 0.000 | 0.257 | -0.020 | 0.282 | -0.018 |
| PM1 | alpha-D-Lactose | 34.082 | 0.000 | 0.478 | 0.201 | 0.524 | 0.224 |
| PM1 | Lactulose | 20.069 | 0.000 | 0.483 | 0.206 | 0.434 | 0.134 |
| PM1 | Sucrose | 7.677 | 0.000 | 0.612 | 0.335 | 0.551 | 0.251 |
| PM1 | Uridine | 8.055 | 0.000 | 0.309 | 0.032 | 0.329 | 0.029 |
| PM1 | L-Glutamine | 2.638 | 0.000 | 0.273 | -0.004 | 0.308 | 0.008 |
| PM1 | m-Tartaric Acid | 2.104 | 0.000 | 0.277 | 0.000 | 0.284 | -0.016 |
| PM1 | D-Glucose-1-Phosphate | 1.739 | 0.492 | 0.281 | 0.004 | 0.289 | -0.011 |
| PM1 | D-Fructose-6-Phosphate | 2.024 | 0.000 | 0.282 | 0.005 | 0.287 | -0.013 |
| PM1 | Tween 80 | 3.536 | 0.000 | 0.285 | 0.008 | 0.327 | 0.027 |
| PM1 | alpha-Hydroxy Glutaric Acid-gamma-Lactone | 1.552 | 0.000 | 0.277 | 0.000 | 0.307 | 0.007 |
| PM1 | alpha-Hydroxy Butyric Acid | 1.133 | 0.000 | 0.278 | 0.001 | 0.315 | 0.015 |
| PM1 | beta-Methyl-D Glucoside | 2.216 | 0.000 | 0.276 | -0.001 | 0.302 | 0.002 |
| PM1 | Adonitol | 2.216 | 0.290 | 0.279 | 0.002 | 0.301 | 0.001 |
| PM1 | Maltotriose | 3.352 | 0.000 | 0.311 | 0.034 | 0.318 | 0.018 |
| PM1 | 2-Deoxy Adenosine |  | 0.000 | 0.380 | 0.103 | 0.387 | 0.087 |
| PM1 | Adenosine |  | 0.000 | 0.375 | 0.098 | 0.360 | 0.060 |
| PM1 | Glycyl-L-Aspartic Acid | 0.165 | 2.765 | 0.266 | -0.011 | 0.264 | -0.036 |
| PM1 | Citric Acid | 1.330 | 2.871 | 0.277 | 0.000 | 0.281 | -0.019 |
| PM1 | m-Inositol | 2.353 | 9.086 | 0.275 | -0.002 | 0.284 | -0.016 |
| PM1 | D-Threonine | 1.454 | 0.597 | 0.280 | 0.003 | 0.292 | -0.008 |
| PM1 | Fumaric Acid | 2.202 | 0.000 | 0.282 | 0.005 | 0.287 | -0.013 |
| PM1 | Bromo Succinic Acid | 1.641 | 1.238 | 0.272 | -0.005 | 0.275 | -0.025 |
| PM1 | Propionic Acid | 1.731 | 2.888 | 0.289 | 0.012 | 0.286 | -0.014 |
| PM1 | Mucic Acid | 2.471 | 0.000 | 0.291 | 0.014 | 0.312 | 0.012 |
| PM1 | Glycolic Acid | 2.392 | 0.000 | 0.279 | 0.002 | 0.282 | -0.018 |
| PM1 | Glyoxylic Acid | 2.982 | 0.000 | 0.290 | 0.013 | 0.282 | -0.018 |
| PM1 | D-Cellobiose | 2.727 | 0.000 | 0.279 | 0.002 | 0.295 | -0.005 |
| PM1 | Inosine |  | 0.000 | 0.376 | 0.099 | 0.370 | 0.070 |
| PM1 | Glycyl-L-Glutamic Acid | 0.000 | 2.897 | 0.259 | -0.018 | 0.275 | -0.025 |
| PM1 | Tricarballylic Acid | 2.015 | 1.782 | 0.285 | 0.008 | 0.287 | -0.013 |
| PM1 | L-Serine | 1.463 | 0.000 | 0.277 | 0.000 | 0.278 | -0.022 |
| PM1 | L-Threonine | 1.979 | 4.231 | 0.275 | -0.002 | 0.280 | -0.020 |
| PM1 | L-Alanine | 1.535 | 2.862 | 0.278 | 0.001 | 0.285 | -0.015 |
| PM1 | L-Alanyl-Glycine | 0.645 | 1.510 | 0.267 | -0.010 | 0.268 | -0.032 |
| PM1 | Acetoacetic Acid | 1.626 | 1.176 | 0.308 | 0.031 | 0.295 | -0.005 |
| PM1 | N-Acetyl-beta-D Mannosamine | 1.828 | 0.702 | 0.310 | 0.033 | 0.296 | -0.004 |
| PM1 | Mono Methyl Succinate | 1.670 | 0.614 | 0.285 | 0.008 | 0.295 | -0.005 |
| PM1 | Methyl Pyruvate | 1.467 | 1.519 | 0.270 | -0.007 | 0.267 | -0.033 |
| PM1 | D-Malic Acid | 2.683 | 0.000 | 0.278 | 0.001 | 0.282 | -0.018 |
| PM1 | L-Malic Acid | 2.665 | 0.000 | 0.279 | 0.002 | 0.284 | -0.016 |
| PM1 | Glycyl-L-Proline | 0.000 | 0.000 | 0.281 | 0.004 | 0.308 | 0.008 |
| PM1 | p-Hydroxy Phenyl Acetic Acid | 1.828 | 0.913 | 0.268 | -0.009 | 0.274 | -0.026 |
| PM1 | m-Hydroxy Phenyl Acetic Acid | 0.965 | 0.000 | 0.283 | 0.006 | 0.310 | 0.010 |
| PM1 | Tyramine | 0.574 | 1.273 | 0.279 | 0.002 | 0.310 | 0.010 |
| PM1 | D-Psicose | 1.054 | 0.044 | 0.277 | 0.000 | 0.293 | -0.007 |
| PM1 | L-Lyxose | 1.446 | 0.527 | 0.286 | 0.009 | 0.292 | -0.008 |
| PM1 | Glucuronamide | 1.467 | 0.000 | 0.279 | 0.002 | 0.287 | -0.013 |
| PM1 | Pyruvic Acid | 2.013 | 0.044 | 0.278 | 0.001 | 0.280 | -0.020 |
| PM1 | L-Galactonic Acid-gamma-Lactone | 2.242 | 0.000 | 0.290 | 0.013 | 0.308 | 0.008 |
| PM1 | D-Galacturonic Acid | 2.181 | 0.000 | 0.292 | 0.015 | 0.312 | 0.012 |
| PM1 | Phenylethylamine | 2.013 | 0.000 | 0.285 | 0.008 | 0.322 | 0.022 |
| PM1 | 2-Aminoethanol | 2.454 | 0.000 | 0.371 | 0.094 | 0.289 | -0.011 |
| PM2 | Negative Control | 10.271 | 9.596 | 0.286 | 0.000 | 0.291 | 0.000 |
| PM2 | Chondroitin Sulfate C | 13.405 | 8.309 | 0.280 | -0.006 | 0.279 | -0.012 |
| PM2 | alpha-Cyclodextrin | 3.837 | 7.395 | 0.249 | -0.037 | 0.254 | -0.037 |
| PM2 | beta-Cyclodextrin | 8.908 | 8.586 | 0.271 | -0.015 | 0.276 | -0.015 |
| PM2 | gamma-Cyclodextrin | 7.751 | 8.309 | 0.268 | -0.018 | 0.276 | -0.015 |
| PM2 | Dextrin | 45.892 | 9.834 | 0.293 | 0.007 | 0.293 | 0.002 |
| PM2 | Gelatin | 6.064 | 7.402 | 0.268 | -0.018 | 0.271 | -0.020 |
| PM2 | Glycogen | 8.549 | 7.374 | 0.271 | -0.015 | 0.287 | -0.004 |
| PM2 | Inulin | 9.013 | 7.758 | 0.302 | 0.016 | 0.322 | 0.031 |
| PM2 | Laminarin | 10.048 | 7.084 | 0.274 | -0.012 | 0.279 | -0.012 |
| PM2 | Mannan | 4.979 | 6.644 | 0.276 | -0.010 | 0.302 | 0.011 |
| PM2 | Pectin | 9.974 | 12.740 | 0.305 | 0.019 | 0.316 | 0.025 |
| PM2 | N-Acetyl-D-Galactosamine | 8.542 | 8.395 | 0.287 | 0.001 | 0.290 | -0.001 |
| PM2 | N-AcetylNeuraminic Acid | 8.285 | 6.718 | 0.288 | 0.002 | 0.282 | -0.009 |
| PM2 | beta-D-Allose | 6.535 | 9.014 | 0.287 | 0.001 | 0.292 | 0.001 |
| PM2 | Amygdalin | 7.801 | 8.700 | 0.294 | 0.008 | 0.299 | 0.008 |
| PM2 | D-Arabinose | 9.323 | 8.195 | 0.281 | -0.005 | 0.289 | -0.002 |
| PM2 | D-Arabitol | 19.335 | 8.900 | 0.273 | -0.013 | 0.280 | -0.011 |
| PM2 | L-Arabitol | 9.154 | 1.568 | 0.273 | -0.013 | 0.279 | -0.012 |
| PM2 | Arbutin | 9.949 | 8.189 | 0.326 | 0.040 | 0.321 | 0.030 |
| PM2 | 2-Deoxy-D Ribose | 9.245 | 10.146 | 0.283 | -0.003 | 0.282 | -0.009 |
| PM2 | i-Erythritol | 8.466 | 8.170 | 0.274 | -0.012 | 0.274 | -0.017 |
| PM2 | D-Fucose | 10.264 | 1.737 | 0.251 | -0.035 | 0.263 | -0.028 |
| PM2 | 3-0-beta-D-Galactopyranosyl-D Arabinose | 8.392 | 30.654 | 0.364 | 0.078 | 0.354 | 0.063 |
| PM2 | Gentiobiose | 9.392 | 10.215 | 0.278 | -0.008 | 0.287 | -0.004 |
| PM2 | L-Glucose | 8.542 | 9.862 | 0.281 | -0.005 | 0.287 | -0.004 |
| PM2 | Lactitol | 8.374 | 16.436 | 0.384 | 0.098 | 0.378 | 0.087 |
| PM2 | D-Melezitose | 12.673 | 6.166 | 0.311 | 0.025 | 0.332 | 0.041 |
| PM2 | Maltitol | 14.571 | 11.873 | 0.294 | 0.008 | 0.309 | 0.018 |
| PM2 | a-Methyl-D Glucoside | 8.413 | 9.586 | 0.277 | -0.009 | 0.286 | -0.005 |
| PM2 | beta-Methyl-DGalactoside | 12.690 | 51.649 | 0.363 | 0.077 | 0.355 | 0.064 |
| PM2 | 3-Methyl Glucose | 8.218 | 10.324 | 0.289 | 0.003 | 0.281 | -0.010 |
| PM2 | beta-Methyl-D-Glucuronic Acid | 42.542 | 8.657 | 0.278 | -0.008 | 0.271 | -0.020 |
| PM2 | alpha-Methyl-D-Mannoside | 26.813 | 9.228 | 0.277 | -0.009 | 0.271 | -0.020 |
| PM2 | beta-Methyl-D-Xyloside | 51.222 | 7.861 | 0.277 | -0.009 | 0.275 | -0.016 |
| PM2 | Palatinose | 17.909 | 8.919 | 0.283 | -0.003 | 0.291 | 0.000 |
| PM2 | D-Raffinose | 6.051 | 76.894 | 0.464 | 0.178 | 0.495 | 0.204 |
| PM2 | Salicin | 6.911 | 10.691 | 0.310 | 0.024 | 0.312 | 0.021 |
| PM2 | Sedoheptulosan | 7.860 | 10.034 | 0.294 | 0.008 | 0.288 | -0.003 |
| PM2 | L-Sorbose | 5.369 | 8.243 | 0.284 | -0.002 | 0.286 | -0.005 |
| PM2 | Stachyose | 7.119 | 72.426 | 0.416 | 0.130 | 0.434 | 0.143 |
| PM2 | D-Tagatose | 9.540 | 2.689 | 0.262 | -0.024 | 0.253 | -0.038 |
| PM2 | Turanose | 7.389 | 8.142 | 0.294 | 0.008 | 0.325 | 0.034 |
| PM2 | Xylitol | 11.564 | 8.002 | 0.277 | -0.009 | 0.275 | -0.016 |
| PM2 | N-Acetyl-D Glucosaminitol | 55.297 | 6.147 | 0.284 | -0.002 | 0.296 | 0.005 |
| PM2 | gamma-Amino Butyric Acid | 48.017 | 6.981 | 0.274 | -0.012 | 0.280 | -0.011 |
| PM2 | delta-Amino Valeric Acid | 17.784 | 4.939 | 0.261 | -0.025 | 0.268 | -0.023 |
| PM2 | Butyric Acid | 18.240 | 10.595 | 0.280 | -0.006 | 0.275 | -0.016 |
| PM2 | Capric Acid | 9.669 | 0.802 | 0.254 | -0.032 | 0.428 | 0.137 |
| PM2 | Caproic Acid | 9.135 | 6.347 | 0.277 | -0.009 | 0.288 | -0.003 |
| PM2 | Citraconic Acid | 8.403 | 9.072 | 0.281 | -0.005 | 0.282 | -0.009 |
| PM2 | Citramalic Acid | 7.801 | 6.614 | 0.276 | -0.010 | 0.276 | -0.015 |
| PM2 | D-Glucosamine | 8.789 | 5.594 | 0.304 | 0.018 | 0.313 | 0.022 |
| PM2 | 2-Hydroxy Benzoic Acid | 8.739 | 3.679 | 0.296 | 0.010 | 0.317 | 0.026 |
| PM2 | 4-Hydroxy Benzoic Acid | 8.657 | 5.071 | 0.315 | 0.029 | 0.349 | 0.058 |
| PM2 | beta-Hydroxy Butyric Acid | 9.609 | 6.035 | 0.281 | -0.005 | 0.289 | -0.002 |
| PM2 | gamma-Hydroxy Butyric Acid | 10.512 | 5.492 | 0.283 | -0.003 | 0.277 | -0.014 |
| PM2 | a-Keto-Valeric Acid | 11.017 | 4.368 | 0.266 | -0.020 | 0.292 | 0.001 |
| PM2 | Itaconic Acid |  | 5.595 | 0.273 | -0.013 | 0.272 | -0.019 |
| PM2 | 5-Keto-D Gluconic Acid |  | 5.342 | 0.293 | 0.007 | 0.296 | 0.005 |
| PM2 | D-Lactic Acid Methyl Ester | 3.659 | 8.224 | 0.280 | -0.006 | 0.286 | -0.005 |
| PM2 | Malonic Acid | 8.067 | 6.328 | 0.280 | -0.006 | 0.286 | -0.005 |
| PM2 | Melibionic Acid | 9.599 | 9.596 | 0.322 | 0.036 | 0.327 | 0.036 |
| PM2 | Oxalic Acid | 7.435 | 5.004 | 0.307 | 0.021 | 0.312 | 0.021 |
| PM2 | Oxalomalic Acid | 8.769 | 5.404 | 0.290 | 0.004 | 0.295 | 0.004 |
| PM2 | Quinic Acid | 10.647 | 7.709 | 0.283 | -0.003 | 0.278 | -0.013 |
| PM2 | D-Ribono-1,4-Lactone | 7.978 | 5.614 | 0.288 | 0.002 | 0.281 | -0.010 |
| PM2 | Sebacic Acid | 6.719 | 3.600 | 0.278 | -0.008 | 0.286 | -0.005 |
| PM2 | Sorbic Acid | 7.688 | 2.982 | 0.277 | -0.009 | 0.287 | -0.004 |
| PM2 | Succinamic Acid | 8.814 | 6.513 | 0.282 | -0.004 | 0.281 | -0.010 |
| PM2 | D-Tartaric Acid | 9.427 | 6.840 | 0.278 | -0.008 | 0.284 | -0.007 |
| PM2 | L-Tartaric Acid |  | 7.074 | 0.279 | -0.007 | 0.275 | -0.016 |
| PM2 | Acetamide | 5.745 | 7.538 | 0.278 | -0.008 | 0.278 | -0.013 |
| PM2 | L-Alaninamide | 9.599 | 6.814 | 0.281 | -0.005 | 0.286 | -0.005 |
| PM2 | N-Acetyl-L-Glutamic Acid | 8.324 | 7.052 | 0.278 | -0.008 | 0.278 | -0.013 |
| PM2 | L-Arginine | 8.957 | 11.206 | 0.325 | 0.039 | 0.346 | 0.055 |
| PM2 | Glycine | 7.889 | 6.909 | 0.276 | -0.010 | 0.281 | -0.010 |
| PM2 | L-Histidine | 7.662 | 8.119 | 0.315 | 0.029 | 0.318 | 0.027 |
| PM2 | L-Homoserine | 8.491 | 8.226 | 0.280 | -0.006 | 0.285 | -0.006 |
| PM2 | Hydroxy-L-Proline | 10.173 | 6.971 | 0.283 | -0.003 | 0.286 | -0.005 |
| PM2 | L-Isoleucine | 8.052 | 4.584 | 0.280 | -0.006 | 0.288 | -0.003 |
| PM2 | L-Leucine | 5.600 | 8.170 | 0.278 | -0.008 | 0.504 | 0.213 |
| PM2 | L-Lysine | 9.295 | 6.372 | 0.284 | -0.002 | 0.283 | -0.008 |
| PM2 | L-Methionine | 8.715 | 7.674 | 0.266 | -0.020 | 0.280 | -0.011 |
| PM2 | L-Ornithine | 4.252 | 7.214 | 0.284 | -0.002 | 0.281 | -0.010 |
| PM2 | L-Phenylalanine | 4.885 | 9.405 | 0.304 | 0.018 | 0.308 | 0.017 |
| PM2 | L-Pyroglutamic Acid | 4.065 | 9.281 | 0.274 | -0.012 | 0.283 | -0.008 |
| PM2 | L-Valine | 3.126 | 3.289 | 0.267 | -0.019 | 0.268 | -0.023 |
| PM2 | D,L-Carnitine | 7.247 | 8.281 | 0.278 | -0.008 | 0.280 | -0.011 |
| PM2 | Sec-Butylamine | 8.542 | 7.643 | 0.281 | -0.005 | 0.278 | -0.013 |
| PM2 | D.L-Octopamine | 0.000 | 3.713 | 0.307 | 0.021 | 0.325 | 0.034 |
| PM2 | Putrescine | 9.096 | 6.044 | 0.283 | -0.003 | 0.276 | -0.015 |
| PM2 | Dihydroxy Acetone | 6.702 | 3.685 | 0.310 | 0.024 | 0.319 | 0.028 |
| PM2 | 2,3-Butanediol | 7.158 | 6.541 | 0.287 | 0.001 | 0.291 | 0.000 |
| PM2 | 2,3-Butanedione | 3.637 | 6.794 | 0.278 | -0.008 | 0.279 | -0.012 |
| PM2 | 3-Hydroxy 2-Butanone | 8.251 | 7.000 | 0.279 | -0.007 | 0.357 | 0.066 |
